# Interventions Addressing Health Literacy in Cancer Care: A Systematic Review of Reviews

**DOI:** 10.3390/ijerph22020212

**Published:** 2025-02-02

**Authors:** Celine Jeitani, Stephan Van den Broucke, Charlotte Leemans

**Affiliations:** 1 Psychology Sciences Research Institute, Catholic University of Louvain, 1348 Ottignies-Louvain-la-Neuve, Belgium; stephan.vandenbroucke@uclouvain.be; 2 Psychology Sciences Research Institute, 1348 Ottignies-Louvain-la-Neuve, Belgium; charlotte.leemans@uclouvain.be

**Keywords:** health literacy, intervention, review, oncology, hematology

## Abstract

(1) Background: Interventions addressing the health literacy (HL) of people suffering from an illness such as cancer can improve the understanding of the illness and lead to better-adapted behaviors, regarding the participation in cancer screenings, adhering to the complex multimodal therapy, participating in cancer treatment, and self-managing everyday health. This study provides a review of systematic reviews that include intervention articles addressing the HL of patients, healthcare professionals, and/or organizations in cancer, to identify the factors related to their effectiveness, as well as the missing elements, in light of the recent developments in HL research and practice. (2) Methods: A literature search was performed in Embase, Pubmed, PsycINFO, and Science Direct. Existing published reviews of studies targeting the interventions in the oncology domain, and which explicitly mentioned HL as a factor/outcome, were included. (3) Results: One hundred and fifty-five studies were retrieved. Ten fit the criteria and were included in this review. (4) Conclusions: Most of the interventions addressing HL in people with cancer included the target patients’ information and communication skills through education. To keep the full scope of the concept, as investigated in the recent literature, clinical applications of HL in patients with cancer should also consider organizational HL.

## 1. Introduction

Over the last decades, health literacy (HL) has gained critical importance in public health and healthcare [1,2]. Although several definitions of the concept exist [3], HL is generally agreed to refer to people’s knowledge, motivation, and competencies to access, understand, appraise, and apply health information to decision-making in healthcare, disease prevention, and health promotion [4]. This specific competence has become increasingly relevant in the context of patient-centered care, where the patients and their informal carers are actively involved in the decision-making regarding their health in different domains, such as disease prevention and treatment. Informed decision-making not only requires that information is communicated by health professionals or by the health system, but also that it is adapted to each patient’s level of comprehension of medical jargon, and their aptitude for navigating health services [5]. Patients with limited HL have more difficulty navigating through the system and making decisions, especially in high-burden diseases such as cancer [6]. In addition, low HL has been associated with less participation in preventive cancer screening [7] and with more risky and problematic behavior, resulting in poor treatment adherence and poor illness self-management [8]. As such, adequate levels of HL are essential when dealing with and managing chronic illnesses as complex as cancer [9]. Cancer care offers a range of treatment options, varying in duration and complexity, with some being short-term, others long-term, and often requiring a combination of approaches to address a single issue effectively. These treatments can be surgeries, preventative screenings, chemotherapy, radiotherapy, and others, which is why cancer care necessitates a certain type of HL level and care coordination [10].

While emphasizing its role in informed health decision-making in everyday life, the above-mentioned definition of HL by Sorensen et al. [4] recognizes its multidimensional character and its applicability within the healthcare, disease prevention and health promotion setting. This has also been highlighted by other authors. For instance, Nutbeam [11] distinguishes between functional, communicative and critical HL, while Stocks et al. [9] shift the focus from understanding health information in healthcare to motivating health-related actions and Wu et al. (2010) [12] consider the empowerment of health literate individuals in controlling their health behaviors and living conditions as key. Likewise, Dodson et al. [13] consider HL to include a broad range of competencies enabling sound health decisions and proactive engagement with factors that impact health. Freedman et al. [14] stress the communal dimension, defining HL as the ability of not only individuals, but also of groups, to use information for public health decisions. HL is indeed often considered in relationship with health inequities, in the sense that acts as a social determinant of health [15], or as a mediator between social and economic determinants and specific health outcomes, health-related behaviors, and access to health services [16,17].

As a set of competencies linked to general literacy, HL can be conceived of as the product of health education [11]. In that regard, Berkman and colleagues [6] focus on the educational purposes of HL rather than on specific skills such as analysis, filtration of information, application, etc. Not surprisingly, the growing awareness of the role of HL for healthcare, health behaviors, health actions, and health inequalities has been accompanied by efforts to improve HL levels in populations. This has resulted in a large number of interventional studies and strategies targeting limited HL through patient education, health education, and health promotion. Specifically, operationalizing the HL concept in interventions is meant to target a set of functional skills and more complex competencies related to health behaviors, such as self-management, problem-solving skills, decision-making, application, and others [18]. A review of intervention studies by Berkman and colleagues [6] revealed that there is a large variety of intervention types, ranging from single-features, such as one-time information sessions, flyers, booklets, and/or other tools meant to be used by patients for educational purposes, to more encompassing intervention programs targeting self-management, self-efficacy, adherence, and skill building, aimed at behavior change.

In the past years, several reviews have been performed to synthesize the findings from the intervention studies on the quality, outcomes, feasibility, and efficacy of interventions addressing HL [19,20,21]. However, given the variety of intervention types and outcome indicators that were involved in these reviews, the results could not easily be compared, nor could the conclusions be generalized. Therefore, this review of reviews is set to explore the conclusions drawn by the included reviews that have addressed interventions with a HL factor by going over the included studies, the analyses, and the role HL played in the theory or application of the interventions. Exploring the above could allow for a better understanding of the position of HL in what is considered a “health literacy intervention”.

The current review addresses the existing reviews in the form of a meta-review, focusing specifically on the dimensions of HL when considering the results of the interventions concerned. This focus on HL allows for a better understanding of the usage of the term in itself, its role in interventions, and its influence on the results. Specifically, the review will synthesize the existing systematic reviews that study cancer-specific interventional studies including HL, bringing the HL aspect forward through compiling the information from different sources, looking into (1) the features of the different interventions included in existing review studies, (2) the outcomes of existing systematic reviews, (3) the conclusions drawn from the interventional studies in terms of the importance, efficacy, and impact of/on HL, as presented in the existing reviews, and (4) the different aspects of HL brought out in the interventions through the analysis of the reviewers.

## 2. Method

This study is a review of systematic reviews looking into interventions targeting HL as a mediating variable or as an outcome among patients with cancer. The PRISMA (Preferred Reporting Items for Systematic Reviews and Meta-Analyses) declaration and the methodological guidelines for utilizing existing systematic reviews were followed in the conduct of the study [22] (Appendix A). The review protocol was submitted to Prospero in February 2022.

### 2.1. Search Strategy

A literature search was performed using Embase, Pubmed, PsycINFO, and Science Direct. The search string used in performing the literature search focused on 5 themes: health literacy, oncology, interventions, population (adult patients and health professionals), and the nature of the article (review) (can be accessed in Supplementary Materials). Due to the recent developments and peaked interest in HL, articles published before 2010 were not considered. Specifically, HL started being officially recognized around 2009, after a report by WHO “Health Promotion and Health Literacy” [23], in addition to the US department of Health and Human Services launching the “National Action Plan to Improve Health Literacy” [24]. The literature search included articles in French, English, and Spanish. The first literature search wave was conducted in February 2022, with updates in March 2023 and January 2024 in order to include any newly released studies during the review process. The articles that were identified were imported into CADIMA.

### 2.2. Study Selection

**Figure 1:**
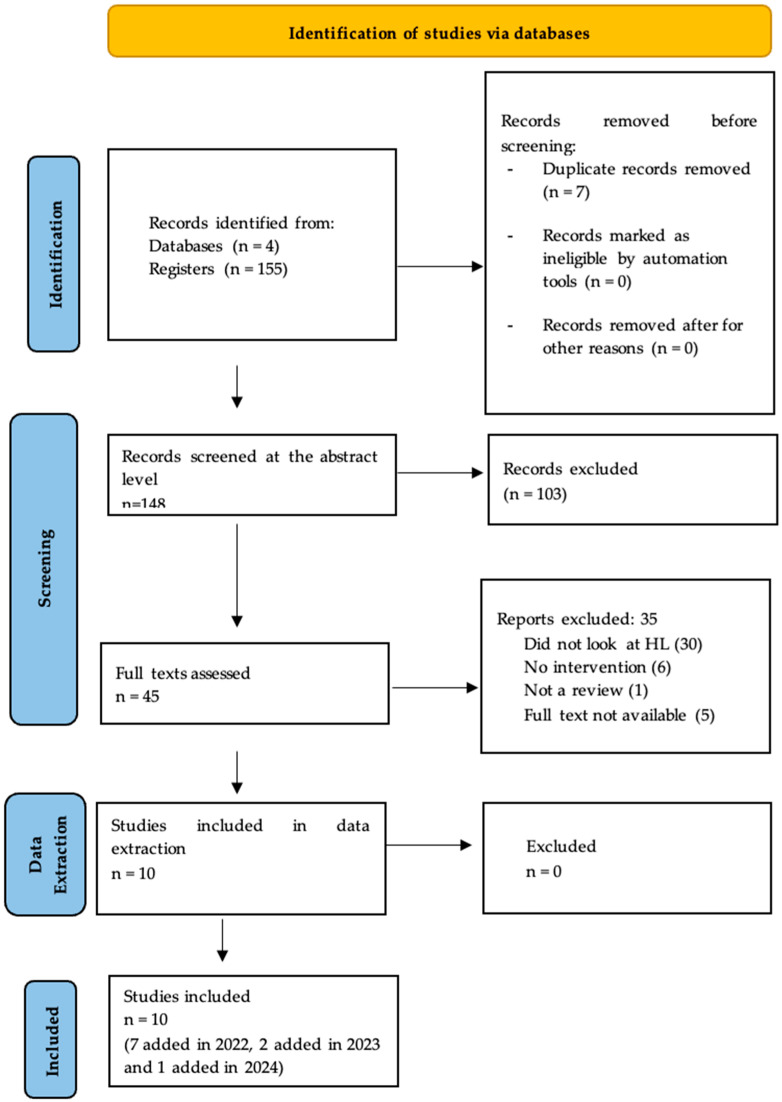
PRISMA Screening Graph 1.

Reviews that studied interventions addressing health literacy within cancer care (targeting organizations, professionals, and/or patients) were included. The inclusion criteria were (1) the article should include a cancer-related population, (2) the review should include interventions targeting adult patients and/or health professionals, (3) health literacy or health competence should be explicitly mentioned. Studies that included health literacy as a targeted outcome, a determinant of intervention effectiveness, a moderator, a mediating variable, used HL-specific tools, or factored in the influence on health-related outcomes were included. Studies could have addressed health literacy as a whole or focused on specific aspects (e.g., comprehension, information use, decision-making, or healthcare-system navigation). First, the titles and abstracts of reviews found through the literature search were screened by two evaluators (CJ and CL), with a third evaluator (SV) solicited in the case of disagreements. The interrater validity between the two evaluators was assured through the repeated testing of 3 random studies and discussions on clarifying the criteria until the evaluators reached full agreement. In the second step, full-text screening was conducted for the reviews that passed the inclusion criteria based on their titles and abstracts. The full texts were also screened by the two evaluators, with a third evaluator involved in the case of opposing results. Comments were also added on the excluded studies, in order to compare notes in cases of potential disagreements. While adhering to the inclusion criteria and screening for intervention studies addressing oncology populations and health literacy, three of the included reviews examined multiple diseases, with cancer being one among them. Consistent with our criteria, these reviews were included, and the data, along with conclusions specific to cancer-related studies, were extracted for the analysis. As a result of the screening process, 10 review studies (out of 148 screened) met the criteria and were considered eligible for data extraction. Figure 1 shows the PRISMA flow diagram representing the flow of information through the different phases of a systematic review. 

### 2.3. Data Extraction

Data extraction was performed by two individual reviewers (CJ and CL), using a data extraction form created to fit the characteristics evaluated in this review. Four separate sheets were created, each concerning a different aspect of each systematic review: (1) general information about the studies (type of studies, populations, criteria, countries, etc.); (2) quality data; (3) the content of the interventions included in each review (type of intervention, frequency, tools, persons involved, etc.); and (4) outcomes.

### 2.4. Quality Assessment and Risk of Bias

A quality assessment of the 10 review articles included in the paper was performed using a systematic review and a meta-analysis assessment tool from the National Institute of Health (NIH), specifically the National Heart, Lung, and Blood Institute’s (NHLBI). The criteria of this tool assess the review’s objectives, including a well-formulated question and pre-defined inclusion and exclusion criteria. It scrutinizes the process of the literature search, the screening process, and the thoroughness of the evaluation of each study’s quality. Additionally, the criteria consider transparent reporting of included studies, potential publication bias, and heterogeneity in meta-analyses, when applicable. Each of the 8 criteria was assessed by two reviewers (CJ and CL). Similar to the screening process, a third reviewer (SV) was involved in case of inconsistencies. For each criterium, a score was given corresponding with the categories Good, Fair, Poor, Not Applicable, Cannot Determine, and Not Reported. The majority of the studies scored ‘good’ on the first three criteria and on eligibility criteria: clear questions, screening process, and study presentation. The rest varied. A table with details regarding the quality scores of each article is given in Appendix B. The studies’ overall quality can be characterized as ranging from fair to good. For a deeper analysis, the risk of bias was assessed through the ROBIS tool [25]. The reviews scored between moderate- and low-risk. No study scored a high risk of bias. All study objectives, included articles, and methodologies were aligned. The ROBIS table can be found in the Supplementary Materials. Most studies used databases in the search strategy. An overlap assessment was performed (Appendix C). Reviews Hill et al. (2) [26] and Mustermann et al. (9) [27] had the most overlap, with 12 articles overlapping between each other. This can be explained through the specificity of the population of both articles (deaf patients with cancer). There was a difference in the analysis of results, as Hill et al. focused more on barriers and health disparities in the interventions whilst Mustermann et al. focused more on the interventions and their outcomes. Two meta-analyses are included in the reviews (4,10) [28,29].

## 3. Results

Table 1 shows a summary of the basic characteristics of each of the 10 review articles that were included.

### 3.1. Study Types

The review papers included in this review included between ten (7) [20] and fifty-three (4) [28] primary studies. The total number of participants was not always clear. They included interventional studies, surveys, and simple comparative studies focusing on HL in the context of oncology. However, they all included at least one interventional study that targeted at least one of the aspects of HL (e.g., informed decision-making). The intervention studies varied from testing the efficacy of online interventions to analyzing the importance of promoting certain aspects such as decision-making, competence, and patient education.

### 3.2. Types of Cancer

Although none of the reviews in this study targeted breast cancer specifically, breast cancer was the most often studied type of cancer, and was represented in all ten the reviews. The reviews by DeRosa et al. (3) [31] and by Fernandez-Gonzalez and Bravo-Valenzuela (7) [20] were concerned with HL in breast and prostate cancer, while the ones by McAlpine et al. (1) [30], Housten et al. (5) [21], and Cabanes et al. (8) [33] had 50%, 40%, and one-third of their study population suffering from breast cancer, respectively. As mentioned above, three of the review studies (4,6,10) [28,29,32] included cancer as one of several non-communicable diseases. The review by Heine et al. (4) [28] included only one interventional study that targeted patients with cancer; in the interventional studies included in the review by van der Kruk et al. (6) [32], half were concerned with cancer; and the review by Verweel et al. (10) [29] contained seventeen studies on chronic illnesses, four of which focused specifically on cancer, and two of which included cancer along with other diseases. In the review by Verweel et al. (10) [29], six of the studies were concerned with cancer, three of which were specifically focused on breast cancer. Other types of cancer, such as prostate and colorectal cancer, were highly present.

### 3.3. Participants

Most of the reviews focused on HL among individual adult patients, with an average age ranging from 42 to 70 years. However, the review by Housten et al. (5) [21] also included the HL of peers and caregivers. DeRosa et al.’s review (3) [31] also mentioned the importance of including family and peers (social support) in education and decision-aid of patients with cancer, as they play a crucial role in addressing HL. The review by Hill et al. (2) [26] also included interventions involving health professionals, since they fit into their target of building patient-specific culturally competent care. Four of the reviews (2,3,7,9) [20,26,27,31] targeted minority populations, notably deaf patients (2,9) [20,31] and African, Hispanic, Latin, or Asian populations (3,7) [26,27].

### 3.4. General Description of Interventions

Almost all the reviews included studies involving primarily online interventions or online assistance for face-to-face interventions. Five reviews (1,6,7,9,10) [20,27,29,30,32] were concerned with studies that were completely online, while the others (2,3,4,5,7) [20,21,26,28,31] considered mixed methods, such as face-to-face interventions (group or individual), interviews, etc. The interventions that did not occur online, such as interviews (2), educational programs (1,2,3,4,6,7,8) [20,26,28,31,32,33], information sessions (2,3,4,7,8) [20,26,28,31,33], workshops (3,5,6,8) [21,31,32,33], and handouts (4,5) [21,28], were mostly delivered by the research team, health educators, or medical staff such as nurses, pharmacists, and/or social workers. The review by Verweel et al. (10) [29] focused specifically on digital health literacy, and therefore included solely digital tools, whether internet-based or using other digital means (such as computer software, smart devices, websites, learning management systems, and electronic personal health records (ePHR)), while study 6 [20] had a virtual reality approach. Educational videos were the most commonly used approach to address health literacy, featured in at least one intervention across all ten reviews.

The subjects of the educational interventions varied from specific topics, such as fatigue, insomnia, fertility, diet, and smoking cessation, to more general topics, involving cancer-related knowledge, screening information, and general symptoms. Other interventions included coping skills training, communication skills training (for professionals), symptom monitoring and self-management, self-care, treatment adherence, and decision-making. While these programs are clearly linked to HL, the reviews did not provide enough detail about the content to clearly define this connection. Nevertheless, it is clear that education, digital or not, is the main tool to address HL among patients with cancer.

### 3.5. The Role of Health Literacy

#### 3.5.1. Operationalization of HL

The outcomes of the reviews comprised in this review are shown in Table 1. All reviews included in this study involved one or more interventions, and, in accordance with the inclusion criteria, had to focus on HL as a theme within the interventions to be included in the study. While all of them analyzed the impact of certain factors related to HL, not all necessarily considered HL itself as a measured outcome. Other outcomes that were measured were decision-making, adherence, knowledge, understanding, communication, self-efficacy and self-management, which are related to HL, or a part of its definition. In some studies, however, the outcomes were less directly related to HL, such as quality of life (QoL), mood, physical symptoms, support, coping, PTSD, pain, hope, sleep, or motivation. Moreover, the measurement of HL and related variables varied greatly across the studies, with only a few conducted using thorough evaluations and specific scales to measure HL, such as the Health Literacy Questionnaire (HLQ), the Health Literacy Survey questionnaire (HLS-Q), the Short Test of Functional HL (STFHL), the Rapid Estimate of Adult Literacy in medicine, or the eHealth Literacy scale. Given also that some interventions did not concretely assess HL, determining the intervention’s effect on HL levels in some reviews remains unclear and heterogeneous, making it difficult to draw definitive conclusions.

#### 3.5.2. Health Literacy as an Outcome

Of the ten reviews included in this study, only four contained studies that explicitly considered HL as an intervention outcome. Of these, two remain rather general concerning the operationalization of HL: Heine et al.’s (4) [28] review clearly mentions HL as an outcome but does not specify how it was operationalized in the studies included in their review. Housten et al. (5) [21] mention that the interventions, which aimed to improve specific aspects of HL, led to a significant improvement in two interventions [34,35], but that the outcomes varied depending on whether the baseline level of HL was limited or adequate. The review by Fernandez-Gonzalez and Bravo-Valenzuela (7) [20], via various questionnaires, measured HL as an outcome, along with other factors, such as self-efficacy, motivation, etc. The fourth review by Verweel et al. (10) [29] is rather specific, in the sense that it looked at HL interventions among patients with cancer through digital media. Only one study in this review measured HL as an outcome, while the other ones measured specific skills such as ‘cancer competence’. Interestingly, the one that considered digital health literacy as the outcome [36] showed a significant improvement in comparison to the control group, whereas the other four studies yielded contradictory evidence, with only some of them showing significant change in competence levels, compared to the control groups. The review by McAlpine et al. (1) [30] also describes health competence as an outcome of education interventions, but does not provide any further information on the measurement, impact, or implications. The review included only one study [37] that explicitly targeted health competence, measured via an 8-item questionnaire, but found no significant improvement. The review by Hill et al. (2) [26] mentions the impact of the different interventions on the levels of HL without detailing the operationalizations of HL and finding mixed evidence of efficacy for online interventions, while DeRosa et al. (3) [31] concluded that in most of the studies they reviewed, HL increased as a result of the introduction of navigators that help and support decision making. While ‘better HL awareness’ was identified as a positive decision-making outcome, there was no mention of HL measurement. Van der Kruk and colleagues (6) [32], who reviewed the impact of using virtual reality (VR) in patient education, reported that most participants had a low baseline HL level, which improved as a result of the interventions, but no measurements of HL were mentioned. Finally, the review by Munstermann et al. (9) [27] used the term ‘HL interventions’ to refer to educational interventions and evaluated the impact of these interventions on the participants’ HL levels, but again, does not specify which specific HL measurement methods were used.

#### 3.5.3. Health Literacy as a Moderator

Contrary to the reviews that considered HL as an intervention outcome (4,5,9) [21,27,28], some reviews looked at the moderating effects of HL (8) [33]. Some of these reviews also considered HL as an intervention outcome, and although the specificities regarding to the measurement of the potential moderating impact of HL were mostly unclear, the analyses and conclusion that are drawn by the reviewers suggest that HL is mainly seen to have a moderating effect. Specifically, low HL is seen to act as a barrier preventing the access to population-appropriate healthcare, while higher HL facilitates access to care and affects patient–physician relationships positively. Munstermann and colleagues (9) [27] reported that lower HL was related to inequalities and the inaccessibility of appropriate care in deaf and hard-of-hearing patients, influencing the effect of educational interventions on cancer-knowledge and quality of life. On a similar note, the review by Cabanes et al. (8) [33] considers HL as a means of ‘supportive care’, allowing for a more positive impact on the quality of life and on the reduction in the ‘burden’ of cancer. While these two reviews consider the moderating effect of HL indirectly, the reviews by Heine et al. (4) [28] and Housten, et al. (5) [21] are more explicit about the moderator role of HL on the effects of interventions. The first identifies HL as a moderator of the effects of educational interventions with patients with cancer regarding lifestyle and dietary changes, whereas the second explicitly uses the term ‘modification’ to describe how HL influenced the effects of the interventions with patients with cancer regarding screening and tasks such as recall and recognition.

#### 3.5.4. Effects of HL Interventions on Other Outcome Variables

In addition to being an outcome of an educational intervention, or a moderator of its effects on other outcome measures, HL can also be considered the main theme of an intervention, the effects of which are then assessed via other variables. The review by DeRosa et al. (3) [31] concluded that interventions aiming to enhance ‘HL awareness’ resulted in more satisfaction and self-efficacy, which are in turn linked to decision adherence. Heine et al.’s (4) [28] review, which considered various non-communicable diseases, showed an increase in knowledge, attitude (self-efficacy, motivation, etc.), and self-management behavior, albeit more so among diabetes patients than among patients with cancer. The review by Verweel and colleagues (10) [29] mentions an effect of digital HL interventions among patients with cancer on other outcome variables. Van der Kruk and colleagues (6) [32], who reviewed the impact of using virtual reality (VR) in HL-based patient education, found a significant improvement of knowledge, comprehension, and understanding in most of the studies. The review conducted by Fernandez–Gonzalez and Bravo–Valenzuela (7) [20] showed correlations between HL and other variables such as self-care, knowledge, self-efficacy, and adherence, although the role of HL within those links was not made very clear. Finally, in 6 of the 35 articles reviewed by Cabanes et al. (8) [33], HL-based interventions had an overall positive effect on the QoL, which showed a significant improvement. However, while it is possible for the above-mentioned factors to have a link to HL, the diversity in the types of interventions and outcome measures and the lack of clarity regarding their measurements make it difficult to draw conclusions regarding the actual effect of HL on these variables.

#### 3.5.5. Interventions

The included reviews included various interventions, which tackled similar objectives with comparable strategies. Online interventions showed positive effects on patients with cancer; however, their significance was questioned, as the findings could’ve been affected by the outcome measures (1) [30]. ‘Online’ interventions included platforms linking patients with clinics (and even with other patients) (1) [30], videos with illness-specific information, surveys, media, and talk sessions (Table 1). Technology was said to have limited effects if administered alone, and should rather take various, accessible approaches (10) [29]. Accessibility was thoroughly discussed and highlighted in most of the reviews included, especially through tailored interventions. Tailored interventions showed better results, whether the tailoring relates to the type of patient, to the type of cancer, or even to each individual; however, one review (10) [29] that included results of cancer-specific interventions showed less significant results than, for example, HIV-, diabetes-, or COPD-specific interventions. The advantage of modifying said interventions to the person’s needs, and targeting each patient individually, was the conclusion of not only each intervention included in the reviews, but also by the reviewers of each review included (1,2,3,4,5,7,8,9,10) [20,21,26,27,28,29,30,31,33]. Contextual appropriateness was also considered in the interventions; whether regarding age, culture, specific needs, etc. (2,3,4) [26,28,31]. Finally, multilevel interventions showed better results, in the few studies where they were attempted (5,8) [21,33].

### 3.6. Conclusions

The authors of the ten reviews drew different, yet concurrent, conclusions from their analysis. The mixed results that were reported after the interventions included in the reviews were partly attributed to the weak theoretical basis and weak operational definitions of HL used in many of the studies. Most studies considered HL as an idea, a concept or, at best, as one of the outcomes, but rarely as the primary one. Another point that was raised in several of the reviews (4,5,7,8) [20,21,28,33] is the fact that HL, as a complex concept, is often approached holistically, rather than being broken down into its various dimensions for a deeper understanding and a more precise targeting. Moreover, the reported outcomes varied largely depending on the type of interventions, the target population, and the frequency, duration, and timing of the interventions (1) [30], which makes generalizable interventions more difficult. Tailored interventions were believed to be more effective, as shown in the studies involving deaf patients (2,9) [26,27], at-risk groups (3,7) [20,31], and specific type of cancers (7) [20]. It was also pointed out that the effectiveness of HL interventions highly depends on the availability and accessibility of the resources that are required to implement them at different levels of care (prevention, screening, interventions) (4) [28], and that individual needs, disease-specific information, and preferences must be accounted for when designing HL interventions (10) [29]. The integration of technology and education, delivered by trusted sources, was believed to be effective, even though no significant effects were reported in cancer-specific interventions. Stand-alone technology interventions had limited effects, while the education-based interventions combined with the technology-based approaches showed more promise (10) [29].

## 4. Discussion

This review of reviews summarized the existing systematic reviews and meta-analyses of published studies investigating HL as an outcome, a moderator of outcomes, or a component of interventions in patients with cancer. Although all the reviews included in the study mention HL as a part of the interventions, the role of HL varies significantly between the reviews. Many reviews saw HL as an outcome, even though the outcome measurement was not always appropriate, clearly defined, or detailed. Others considered HL as a moderator, but the measurement methods are unclear. Some reviews considered HL as both an outcome *and* a moderator, and some saw it mainly as a type or a component of an educational intervention with patients with cancer. The majority of interventions focused on adult patients, but some also included peers, family, or healthcare providers. The interventions in the studies varied widely in terms of format, but several involved an online component, and some included VR and eHealth. Other than HL or outcomes moderated by HL, the most commonly used outcomes measured were knowledge, adherence, attitudes (self-efficacy), self-care, and decision-making-related variables.

The reviews pointed to the importance of tailored interventions. Contextual appropriateness as well as individual needs were themes that emerged frequently and could fall under the umbrella of ‘tailored’ interventions. Sudore and Schillinger [38] stress the importance of tailoring communication to the patient’s perceived barriers and their needs for better quality interactions. Brooks and colleagues [39] took a different approach by showing the importance of building trust to improve the health literacy of elderly patients. This was conducted through more thought-out, tailored interventions that met individual needs. According to Salter et al., [40] HL levels vary depending on the healthcare system requirements and a person’s particular skills. However, in elderly patients, while the difficulty of dealing with online tools was recognized in discussions of the reviews, it was not specified how to adapt these digital tools to the population in question, or even the part of the population that does not have access to digital tools. Also, DeMarco and Nystrom [41] emphasized the adaptation of patient education tools to patient-specific needs, which led to positive results. Therefore, the importance of intervening with tools that are adapted to the individual’s existing strengths and limitations is appears in the more positive results of tailored interventions.

Nevertheless, tailored health literacy interventions require an exhaustive understanding of HL as a concept. In the articles included in this review, authors pointed out the weak understanding of the implementation and consideration of the different dimensions of HL, in addition to weak reported correlations between the factors measured as outcomes and their relation to HL, and the role that HL plays sometimes in moderating those effects. Definitions of HL have pointed out several aspects to be targeted when addressing a HL intervention. Sorensen et al. [42] explain HL through a set of cognitive and behavioral skills needed to make decisions and apply health information. Nutbeam [11] also underlined the social skills needed for HL and pointed out its different levels. Those different levels were to be taken into consideration within the interventions, therefore categorizing health literacy interventions into functional, interactive, and critical aspects, and focusing on the clinical settings, emphasizing the efforts to enhance health literacy among healthcare professionals and simplify healthcare organizations. In his review with Llyod [15], they explore interventions for community populations, underscoring the importance of transferable health literacy skills and ensuring accessibility to different populations. The review advocates for a shift in intervention focus towards improving communication quality, developing transferable skills, and prioritizing interventions for populations disproportionately affected by low health literacy.

In a systematic review by Liu et al. [43] the concepts that emerged from reviewing HL definitions were health decisions, functioning in a healthcare environment, promoting and maintaining health behavior, understanding, and gaining access to healthcare. Those concepts were further explored in Liu’s analysis and showed that every aspect of HL had many factors relating to the patient himself, the healthcare practitioners, and the healthcare system itself. For example, Liu and colleagues mentioned knowledge of health information as not only knowing the information but understanding the terms, being able to discern the relevant information, contextually adapting the information received, and using the information relevant to oneself which is linked to accessing the appropriate resources. In this review, the interventions included looked at the retention of information given in the intervention, but no testing of the usage, management, and processing of the information contextually was conducted clearly. The focus was mainly on giving out the information in an adapted manner, which is a part of implementing HL practices; however, the impact, implementation, management, and application of this information was either rarely measured or vaguely reported. On the other hand, decision-making was evaluated in some interventions following decision-aid procedures; however, no explicit link was made with the type of skills required for these decisions to be taken. Maintaining health through management and partnership with the health institutions was also pointed out in several HL definitions; communication with healthcare professionals appeared in some of the reviews included, but still, no measurement of the impact of that communication was measured in the long-term. The above results show a robust framework concerning HL in most cases, addressing the appropriate questions concerning education, decision making, increasing HL; however, the application and implantation of those practices, as well as the adapted clear measurement of its results and correlations, were unclear, insufficient or/and, in some cases, non-existent. The following lead to an incomprehension of how the definitions and frameworks, relating to understanding and increasing HL, are used, leading to inconclusive results for the most part. The authors pointed out that most of the interventions included were emerging and novel interventions that need further testing and analysis. An important point to cover is the long-term effect of the interventions. Interventions either did not measure long-term effects, or they did, which ended up showing a lack of effects being retained. This outcome can be attributed to the lack of interventions focusing on self-management and the acquisition of skills needed to maintain health.

Another point this outcome can be attributed to is the complexity of the healthcare system that requires constant interventions in order to accompany the patient. Parnell et al. [44] explained the importance of redefining HL as a complex concept, not only relating to the patients’ capacities, but also the healthcare system’s demands and resources. The organization plays a role in adapting to their patients’ HL levels, which can create better environments that promote self-efficacy, self-management, and a better understanding and application of health information [15,45]. No intervention included in this review targeted organizational aspects that would allow for better HL outcomes, limiting the spectrum of HL interventions to mostly the knowledge and information aspects of HL. Kaper and colleagues [46] studied the effectiveness level of OHL interventions at the different (patient, professional, and organization) levels. The review found promising results on the patient level, and intermediate outcomes on the professional and organizational levels. Recent studies are just starting to understand the importance of OHL interventions’ impact, but despite the advancements in the understanding, evidence and implementation remain weak. Kaper and colleagues [46] called for a deeper assessment of the outcomes and development of reliable measurements for a comprehensive analysis. In addition, a longitudinal study conducted by Kaper and colleagues [47] showed how the involvement of organizations and professionals helped to identify the implementation barriers, which led to more positive, long-term results in the assessment of OHL intervention implementation in Irish and Dutch hospitals. This information correlates with an observation in one of the reviews included (2) [26], which explains that inadequate HL poses as a barrier for patients with hearing difficulties, but that the responsibility lies with the organization. Access to appropriate care within institutions, in this case specifically the access to interpreters, adapted healthcare models, and linguistically and culturally competent providers, plays a huge role in breaking that barrier and allowing patients with all levels of HL to benefit from standard care, which will in turn increase the QoL. The review also called for the better training of professionals and for the creation of acceptable standards of health information delivery for different backgrounds.

Liu et al. [43] and Parnell et al. [44] demonstrated the main aspect of HL interventions, primarily focusing on providing knowledge and information. In addition, as established in this review, most interventions did not measure HL itself as an outcome, but rather factors such as the QoL, self-efficacy, decision-making, etc. Even though factors such as self-efficacy appeared to be pertinent moderators/mediators of HL levels, and vice versa, and factors such as the QoL have proved to be impacted by HL levels [48,49,50,51], HL was not always clearly measured to check for impact and/or correlation. Only one study, by Verweel and colleagues (10) [29], correctly defined and included studies that, at least partially, mentioned and/or targeted HL within its role as an outcome or as a moderator. Even though it is unclear whether the articles included in the review that date from 2011 to 2022 correctly defined HL, digital HL, or competence, the review itself adopts an appropriate approach to the term. In one of the review’s studies, a health literacy assessment tool was employed, but it demonstrated no discernible impact. On the contrary, when various cancer-competence tools were utilized to gauge the intervention’s effectiveness—primarily employing illness-specific information tools—significant results were consistently observed. This might suggest that the use of a health literacy assessment might have led to insignificant findings, in contrast to the more targeted and specific cancer-related information competence questionnaires. Therefore, this calls not only for a better and more correct application of HL, based on its theoretical and practical framework, but also for the use of appropriate assessment tools based on what is being sought out during an intervention. On another note, although the patients’ digital education was a recurrent theme in that review, the recency of the review (2024) could attest to a better application and interpretation of HL, with recent focus on the subject.

This unclear application and implementation of HL practices could be interpreted as an overuse of the term HL, particularly in contexts that are strictly focused on knowledge, education, and communication. This situation may also reflect a weak understanding of the term, due to its multidimensional and complex nature. A more suitable approach would be to specify the aspects that need to be implemented, especially if the goal is to focus on and enhance only one aspect of HL, and then to correctly assess the results, in order to understand its specific impact on the targeted variable, and to see if there a ripple effect would occur. Although the evaluated studies frequently included health literacy in patient education, they also frequently did not provide a thorough examination of its more complex aspects. Due to their focus on the particular needs of the groups and the provided targeted strategies, content, and tailored interventions that targeted particular populations—like migrant women or the deaf—showed greater effectiveness. However, there is little information available on the long-term results, making it difficult to pinpoint the exact effects and goals of the intervention components. Most interventions were short-term and direct, fulfilling the immediate goals of patient education, but perhaps being less successful in high-stress scenarios like cancer care. Furthermore, it was observed that one-shot therapies were time-consuming and frequently dependent on outside assistance, which made their incorporation into current system less feasible. Instead of only improving the patients’ health literacy skills, organizational health literacy interventions could help them manage their illnesses more effectively through improved self-management and an adjustment to the patients’ needs.

## 5. Clinical Implications

The above-discussed remarks call for suitable application and assessment of health literacy when performing or applying interventions specific to HL or having a HL component. The use of self-efficacy measures or testing information received during an intervention do not reflect on HL in itself. The links between HL and the other components measured (quality of life, self-efficacy, satisfaction, decision-making) can be highlighted and explained to better map out the effects and outcomes of the interventions directly related to HL. In other words, a clearer portrayal of the role of HL (outcome, moderator, mediator) is crucial when presenting the findings. The results of the review also called for tailored interventions, as those show better results and are more accessible to minor populations. Furthermore, better results have also been found when using population-specific instruments, such as cancer-specific HL scales and tests. Finally, the outcomes of this review draw attention to the need for healthcare organizations to consider incorporating accessible interventions, tailored to individual needs, within oncology departments, as a way to render HL more accessible and to emphasize the responsibility of the organization to facilitate access to HL services, rather than focusing solely on educating the patient, which has shown to have little to no long-term effects. A call for a more multilevel, interventional approach, focusing on the three levels: organization, professionals, and patients, is indispensable, considering the different outcomes and the recent emerging theories.

## 6. Limitations

The inclusion criteria explicitly mentioned HL as an outcome or a moderator; however, many of the studies included, despite mentioning HL, did not measure it. This can be considered a limitation, since many other reviews, that included similar interventions which did not mention HL, but measured the same outcomes, were excluded. Therefore, being stricter with the term ‘HL’ and its use within the reviews would allow for a more specific review, solely focused on HL, rather than its simplified interpretations. Three languages were included, but only English search results were found, limiting the scope of the investigation. Standardized measures were not used across the studies, making it difficult to perform a meta-analysis. While we aimed to provide a comprehensive synthesis of the available evidence, the absence of a formal heterogeneity assessment and a meta-analysis prevented us from quantifying the degree of variability among the included studies. This limitation implies that the observed results should be interpreted with caution, and that the generalization of findings in diverse populations or settings may be influenced by a potential heterogeneity. Future research should strive to incorporate robust measures of heterogeneity to enhance the reliability and validity of the meta-analytic findings. This would contribute to a more nuanced understanding of the variability in study outcomes and strengthen the overall quality of evidence in the field.

## 7. Conclusions

This review encourages adequate applications based on the definition of HL, extending beyond the sole focus on communication and education into targeting interventions on multiple levels of HL through mixed-method interventions. Furthermore, the positive outcomes of interventions involving healthcare professionals and peers call for changing the mindset that HL is solely the responsibility of patients, and that it instead addresses the different levels such as patients, environment, professionals, and organizations. Lastly, it addresses the need to use explicit measures of HL as a primary outcome or a moderator when the interventions’ target is HL, in addition to utilizing population-specific strategies and instruments.

## Figures and Tables

**Table 1:** Summary of characteristics of included studies (only the cancer-related studies were taken into account in the mode of delivery, deliverer, measures related to HL, other factors measured and outcome).

Authors and Year	Aim	N of Studies	N of Participants	Mode of Delivery	Deliverer	Measures Related to HL	Other Factors Measured	Outcome
1. McAlpine et al., 2015 [30]	Efficacy of online interventions in patients with cancer.	14	NR	Web-based	NA	Health competence through three-item scale based on previous results of CHESS intervention	QoL, Mood, Cancer symptoms, Social support, Health status, Coping, Self-efficacy, Distress, Stress, PTSD, Adjustment, Self-rated health change, Pain, Hope, Sleep	Mixed efficacy, no harm, little validation
2. Hill et al., 2020 [26]	Review of culturally competent care for deaf patients to better educate professionals and identify barriers to improve care.	34	NR	Online, Surveys, Educational Programs, Interviews	Medical Staff, medical students, ASL interpreters	NR	NR	Disparities among deaf people populations, HL having a low baseline among deaf population allowing tailored interventions to improve those levels, retention of information following intervention.
3. DeRosa, et al., 2021 [31]	Decision-Making support interventions in patients with breast and prostate cancer from racial and ethnic minorities and how QoL is improved.	10	717	Online, software or paper-based material	No third-party facilitator or deliverer	NR	Decision-making adherence, Understanding, Satisfaction, Disease-related knowledge, Self-efficacy	Decision-making support interventions positive impact on minority population communication and informed decision-making.
4. Heine et al., 2021 [28]	Review health literacy interventions in relation to non-communicable diseases especially in low to middle-income countries that are at higher risk for health illiteracy.	53 (42 in quantitative) (1 on cancer)	NR	In-person or group sessions (some with phone call follow-up and support with material or media)	Mostly research team, nurse, or pharmacist	Unclear	Illness-related knowledge, Self-efficacy, Self-care, Self-management, Medication adherence, Motivation	Positive significant effect of HL interventions, however strong dependency on resources of each setting.
5. Housten et al., 2021 [21]	Identify health literacy interventions for patients with cancer and report evidence on study design and intervention characteristics.	36	179,885 (one study had 163,525 participants)	Mixed modes (interventions, training, computerized program, message system, videos, media, and paper material)	Health educator, online, rest unclear.	Health Literacy Questionnaire (HLQ), Assessment of Health Literacy in Cancer Screening (AHL-C), Rapid Evaluation of Adult Literacy in Medicine (REALM), Rapid Health Literacy in Genetics (REAL-G)	Recall, Recognition, Distress, knowledge, Satisfaction, Decisional conflict, Cancer rehabilitation evaluation system, and Communication	Multilevel interventions highest impact, Improvements in adequate HL in some cases rather than limited and other studies showed the opposite result (an improvement from limited HL baseline)
6. van der Kruk et al., 2022 [32]	Review the existing literature on the use of Virtual Reality as a patient education tool.	18 (9 on cancer)	1048 (376 patients with cancer)	Online	Through headsets, controllers, etc.	NA	Anxiety, Behavioral Distress, Understanding, Satisfaction, Knowledge, Fear	Better understanding, less anxiety, good satisfaction,
7. Fernandez-Gonzalez and P. Bravo-Valenzuela, 2019 [20]	Knowing and describing the effectiveness of interventions aimed at improving the HL of patients diagnosed with cancer.	9	NR	Online, videos, group skill trainings, other material (brochures, handouts, images)	Pharmacists, social workers, personal navigators, study coordinators.	REALM, Decisional conflict scale (low literacy version), Self-created questionnaires,	Satisfaction (decision program), Self-efficacy, Adherence, Knowledge, Self-care,	Decrease in uncertainty, increase in knowledge, correlation of Hl with the level of knowledge, the relationship between HL and level of adherence and self-efficacy. Higher HL linked to a better understanding of different domains of the illness.
8. Cabanes et al., 2022. [33]	Assess the type of supportive care interventions for patients with cancer.	35	Range of 45–140 per study	Online or face-to-face	NR	NR	QoL, Anxiety, and Self-esteem.	Increase in QoL
9. Münstermann et al., 2022 [27]	To analyze if cancer education programs promote health literacy among deaf and hard-hearing patients	16	1865	Online or videos	NA	NR	QoL, Cancer knowledge, Stress, Depression, Coping, Support, Gratitude, Optimism.	Increase in QoL, cancer knowledge, and in the concerned studies decrease in stress, improvement of coping.
10. Verweel et al., 2023 [29]	To determine the effects of digital interventions on HL and skills. More specifically looking at the characteristics of the interventions and their impact on self-management, self-efficacy, and patient engagement.	17 (6 cancer-specific)	4877 (1211 known cancer participants (The four studies that specifically target cancer reported their participants giving a total of 1157 cancer participants (450 participants with breast cancer, 295 participants with breast cancer, 102 participants with breast cancer, 310 participants with prostate cancer), one study that included cancer as part of its diseases reported 54 participants, while the other study including cancer did not report the number of participants according to the reviewers.))	Online	Online, registered nurses, community nurses, study staff,	Cancer information competence, eHealth Literacy scale eHEALS, HLS-14, Perceived computer skills	Coping, Emotional Processing, Social Support, Participation, QoL, Self-efficacy, Portal usage	Four out of the six studies that targeted the cancer population showed a significant effect of the intervention group, which scored higher than the control group on cancer competence, perception of computer skills and eHealth Literacy.

## Data Availability

The raw data supporting the conclusions of this article will be made available by the authors on request.

## Supplementary Materials

The following supporting information can be downloaded at: https://www.mdpi.com/article/10.3390/ijerph22020212/s1, File S1: Keywords for database research, can found by contacting celine.jeitani@uclouvain.be.

## Abbreviations

The following abbreviations are used in this manuscript:

MDPI	Multidisciplinary Digital Publishing Institute
HL	Health Literacy
OHL	Organizational Health Literacy
VR	Virtual Reality
NHLBI	National Heart, Lung, and Blood Institute
CJ	Celine Jeitani
CL	Charlotte Leemans
SVDB	Stephan van den Broucke
NA	Not Applicable
NR	Not Reported
NP	Not Possible

## Appendix A. Prisma Checklist

**Table A1:** Prisma Checklist.

Section and Topic	Item #	Checklist Item	Location Where Item Is Reported
**TITLE**			
Title	1	Identify the report as a systematic review.	Page 1
**ABSTRACT**			
Abstract	2	See the PRISMA 2020 for Abstracts checklist.	Page 1
**INTRODUCTION**			
Rationale	3	Describe the rationale for the review in the context of existing knowledge.	End of Introduction
Objectives	4	Provide an explicit statement of the objective(s) or question(s) the review addresses.	End of Introduction
**METHODS**			
Eligibility criteria	5	Specify the inclusion and exclusion criteria for the review and how studies were grouped for the syntheses.	Method
Information sources	6	Specify all databases, registers, websites, organisations, reference lists and other sources searched or consulted to identify studies. Specify the date when each source was last searched or consulted.	Method
Search strategy	7	Present the full search strategies for all databases, registers and websites, including any filters and limits used.	Method and Supplementary File S1
Selection process	8	Specify the methods used to decide whether a study met the inclusion criteria of the review, including how many reviewers screened each record and each report retrieved, whether they worked independently, and if applicable, details of automation tools used in the process.	Method
Data collection process	9	Specify the methods used to collect data from reports, including how many reviewers collected data from each report, whether they worked independently, any processes for obtaining or confirming data from study investigators, and if applicable, details of automation tools used in the process.	Method
Data items	10a	List and define all outcomes for which data were sought. Specify whether all results that were compatible with each outcome domain in each study were sought (e.g., for all measures, time points, analyses), and if not, the methods used to decide which results to collect.	Method
	10b	List and define all other variables for which data were sought (e.g., participant and intervention characteristics, funding sources). Describe any assumptions made about any missing or unclear information.	Results
Study risk of bias assessment	11	Specify the methods used to assess risk of bias in the included studies, including details of the tool(s) used, how many reviewers assessed each study and whether they worked independently, and if applicable, details of automation tools used in the process.	Methods and Appendix
Effect measures	12	Specify for each outcome the effect measure(s) (e.g., risk ratio, mean difference) used in the synthesis or presentation of results.	NR
Synthesis methods	13a	Describe the processes used to decide which studies were eligible for each synthesis (e.g., tabulating the study intervention characteristics and comparing against the planned groups for each synthesis (item #5)).	Results
	13b	Describe any methods required to prepare the data for presentation or synthesis, such as handling of missing summary statistics, or data conversions.	NA
	13c	Describe any methods used to tabulate or visually display results of individual studies and syntheses.	Results
	13d	Describe any methods used to synthesize results and provide a rationale for the choice(s). If meta-analysis was performed, describe the model(s), method(s) to identify the presence and extent of statistical heterogeneity, and software package(s) used.	NA
	13e	Describe any methods used to explore possible causes of heterogeneity among study results (e.g., subgroup analysis, meta-regression).	NA
	13f	Describe any sensitivity analyses conducted to assess robustness of the synthesized results.	NR
Reporting bias assessment	14	Describe any methods used to assess risk of bias due to missing results in a synthesis (arising from reporting biases).	NA (ROB was performed, explanation in Methods and Appendix)
Certainty assessment	15	Describe any methods used to assess certainty (or confidence) in the body of evidence for an outcome.	Methods and Appendix (not explicitly mentioned as certainty but overlap and ROB)
**RESULTS**			
Study selection	16a	Describe the results of the search and selection process, from the number of records identified in the search to the number of studies included in the review, ideally using a flow diagram.	Methods
	16b	Cite studies that might appear to meet the inclusion criteria, but which were excluded, and explain why they were excluded.	Methods
Study characteristics	17	Cite each included study and present its characteristics.	Results and References
Risk of bias in studies	18	Present assessments of risk of bias for each included study.	Methods and Appendix
Results of individual studies	19	For all outcomes, present, for each study: (a) summary statistics for each group (where appropriate) and (b) an effect estimate and its precision (e.g., confidence/credible interval), ideally using structured tables or plots.	NR
Results of syntheses	20a	For each synthesis, briefly summarise the characteristics and risk of bias among contributing studies.	Results and Appendixes
	20b	Present results of all statistical syntheses conducted. If meta-analysis was conducted, present for each the summary estimate and its precision (e.g., confidence/credible interval) and measures of statistical heterogeneity. If comparing groups, describe the direction of the effect.	NA
	20c	Present results of all investigations of possible causes of heterogeneity among study results.	Appendix C
	20d	Present results of all sensitivity analyses conducted to assess the robustness of the synthesized results.	NR
Reporting biases	21	Present assessments of risk of bias due to missing results (arising from reporting biases) for each synthesis assessed.	NA
Certainty of evidence	22	Present assessments of certainty (or confidence) in the body of evidence for each outcome assessed.	NR
**DISCUSSION**			
Discussion	23a	Provide a general interpretation of the results in the context of other evidence.	Discussion
	23b	Discuss any limitations of the evidence included in the review.	Discussion
	23c	Discuss any limitations of the review processes used.	Discussion
	23d	Discuss implications of the results for practice, policy, and future research.	Discussion
**OTHER INFORMATION**			
Registration and protocol	24a	Provide registration information for the review, including register name and registration number, or state that the review was not registered.	Declaration
	24b	Indicate where the review protocol can be accessed, or state that a protocol was not prepared.	NP
	24c	Describe and explain any amendments to information provided at registration or in the protocol.	Declaration
Support	25	Describe sources of financial or non-financial support for the review, and the role of the funders or sponsors in the review.	Declaration
Competing interests	26	Declare any competing interests of review authors.	Declaration
Availability of data, code and other materials	27	Report which of the following are publicly available and where they can be found: template data collection forms; data extracted from included studies; data used for all analyses; analytic code; any other materials used in the review.	Declaration

From: Page MJ, McKenzie JE, Bossuyt PM, Boutron I, Hoffmann TC, Mulrow CD, et al. The PRISMA 2020 statement: an updated guideline for reporting systematic reviews. BMJ 2021;372:n71. doi: 10.1136/bmj.n71. This work is licensed under CC BY 4.0. To view a copy of this license, visit https://creativecommons.org/licenses/by/4.0/ (accessed on 18 September 2024).

## Appendix B. Quality Assessment

**Table A2:** Criteria of NHLBI.

Criteria
1. Is the review based on a focused question that is adequately formulated and described?
2. Were eligibility criteria for the included and excluded studies predefined and specified?
3. Did the literature search strategy use a comprehensive, systematic approach?
4. Were titles, abstracts, and full-text articles dually and independently reviewed for inclusion and exclusion to minimize bias?
5. Was the quality of each included study rated independently by two or more reviewers using a standard method to appraise its internal validity?
6. Were the included studies listed along with important characteristics and results of each study?
7. Was publication bias assessed?
8. Was heterogeneity assessed? (This question applies only to meta-analyses.)

**Table A3:** Assessment of NHLBI Criteria.

CRITERIA ARTICLE	1	2	3	4	5	6	7	8
Cabanes, A., Taylor, C., Malburg, C., and Le, P. T. D. (2022). Supportive care interventions for cancer patients in low-and middle-income countries (LMICs): a scoping review. *Supportive Care in Cancer*, 1–14. [33]	G	G	G	F	P	G	P	NA
DeRosa, A. P., Grell, Y., Razon, D., Komsany, A., Pinheiro, L. C., Martinez, J., and Phillips, E. (2022). Decision-making support among racial and ethnic minorities diagnosed with breast or prostate cancer: A systematic review of the literature. *Patient Education and Counseling*, *105*(5), 1057–1065. [31]	G	G	G	G	G	G	NR	NA
Fernández-González, L., and Bravo-Valenzuela, P. (2019). Effective interventions to improve the health literacy of cancer patients. *ecancermedicalscience*, *13*. [20]	G	G	G	F	NR	G	NR	NA
Heine, M., Lategan, F., Erasmus, M., Lombaard, C. M., Mc Carthy, N., Olivier, J., … and Hanekom, S. (2021). Health education interventions to promote health literacy in adults with selected non-communicable diseases living in low-to-middle income countries: A systematic review and meta-analysis. *Journal of evaluation in clinical practice*, *27*(6), 1417–1428. [28]	G	G	G	G	G	F	G	NR
Hill, C., Deville, C., Alcorn, S., Kiess, A., Viswanathan, A., and Page, B. (2020). Assessing and providing culturally competent care in radiation oncology for deaf cancer patients. *Advances in Radiation Oncology*, *5*(3), 333–344. [26]	G	G	P	NR	NA	G	NR	NA
Housten, A. J., Gunn, C. M., Paasche-Orlow, M. K., and Basen-Engquist, K. M. (2021). Health literacy interventions in cancer: a systematic review. *Journal of Cancer Education*, *36*, 240–252. [28]	G	G	F	F	CD	G	NR	NA
McAlpine, H., Joubert, L., Martin-Sanchez, F., Merolli, M., and Drummond, K. J. (2015). A systematic review of types and efficacy of online interventions for cancer patients. *Patient education and counseling*, *98*(3), 283–295. [30]	G	G	G	F	NA	G	NR	NA
Münstermann, J., Hübner, J., and Büntzel, J. (2022). Can Cancer Education Programs Improve Health Literacy Among Deaf and Hard of Hearing Patients: a Systematic Review. *Journal of Cancer Education*, 1–13. [27]	G	F	P	G	F	G	NR	NA
van der Kruk, S. R., Zielinski, R., MacDougall, H., Hughes-Barton, D., and Gunn, K. M. (2022). Virtual reality as a patient education tool in healthcare: A scoping review. *Patient Education and Counseling*. [32]	G	G	G	G	NA	G	NR	NA
Verweel, L., Newman, A., Michaelchuk, W., Packham, T., Goldstein, R., and Brooks, D. (2023). The effect of digital interventions on related health literacy and skills for individuals living with chronic diseases: A systematic review and meta-analysis. International Journal of Medical Informatics, 105114. [29]	G	G	G	G	F	F	G	F

## Appendix C. Overlap Assessment

**Table A4:** Overlap Assessment.

Full Citation	Included in Review
Choe S et al. (2009) The impact of cervical cancer education for deaf women using a video educational tool employing American sign language, open captioning, and graphics. J Cancer Educ 24(1):10–15. https://doi.org/10.1080/08858190802665245	Hill et al. (2) [26], Mustermann et al. (9) [27]
Cumberland WG et al. (2018) A breast cancer education program for D/deaf women. Am Ann Deaf 163(2):90–115. https://doi.org/10.1353/aad.2018.0014	Hill et al. (2) [26], Mustermann et al. (9) [27]
Folkins A et al. (2005) Improving the deaf community’s access to prostate and testicular cancer information: a survey study. BMC Public Health 5:63. https://doi.org/10.1186/1471-2458-5-63	Hill et al. (2) [26], Mustermann et al. (9) [27]
Harry KM et al. (2012) Evaluating a skin cancer education program for the deaf community. J Cancer Educ 27(3):501–506. https://doi.org/10.1007/s13187-012-0367-7	Hill et al. (2) [26], Mustermann et al. (9) [27]
Hickey S et al. (2013) Breast cancer education for the deaf community in American Sign Language. Oncol Nurs Forum 40(3):E86–91. https://doi.org/10.1188/13.ONF.E86-E91	Hill et al. (2) [26], Mustermann et al. (9) [27]
Jensen LG et al. (2013) Ovarian cancer: deaf and hearing women’s knowledge before and after an educational video. J Cancer Educ 28(4):647–655. https://doi.org/10.1007/s13187-013-0529-2	Hill et al. (2) [26], Mustermann et al. (9) [27]
Jibaja-Weiss ML, Volk RJ, and Friedman LC, et al. (2006) Preliminary testing of a just-in-time, user-defined values clarification exercise to aid lower literate women in making informed breast cancer treatment decisions. Health Expect 9:218–231 https://doi.org/10.1111/j.1369-7625.2006.00386.x PMID: 16911136 PMCID: 5060365	De Rosa et al. (3) [31], Fernandez-Gonzalez et al. (7) [20]
Jibaja-Weiss ML, Volk RJ, Granchi TS et al. (2011) Entertainment education for breast cancer surgery decisions: a randomized trial among patients with low health literacy. Patient Educ Couns 84(1):41–48	De Rosa et al. (3) [31], Fernandez-Gonzalez et al. (7) [20], Housten et al. (5) [28]
Kaskowitz SR et al. (2006) Bringing prostate cancer education to deaf men. Cancer Detect Prev 30(5):439–448. https://doi.org/10.1016/j.cdp.2006.09.001	Hill et al. (2) [26], Mustermann et al. (9) [27]
Kim S, Knight S, and Tomori C, et al. (2001) Health literacy and shared decision making for prostate cancer patients with low socioeconomic status. Cancer Invest 19:684–691 https://doi.org/10.1081/CNV-100106143 PMID: 11577809	De Rosa et al. (3) [31], Fernandez-Gonzalez et al. (7) [20]
Kushalnagar P, Smith S, Hopper M, Ryan C, Rinkevich M, Kushalnagar R (2018) Making cancer health text on the Internet easier to read for deaf people who use American sign language. J Cancer Educ 33(1):134–140	Hill et al. (2) [26], Housten et al. (5) [28]
Palmer CG et al. (2017) Bilingual approach to online cancer genetics education for Deaf American Sign Language users produces greater knowledge and confidence than English text only: a randomized study. Disabil Health J 10(1):23–32. https://doi.org/10.1016/j.dhjo.2016.07.002	Hill et al. (2) [26], Mustermann et al. (9) [27]
Sadler GR et al. (2001) Bringing breast cancer education to deaf women. J Cancer Educ 16(4):225–228. https://doi.org/10.1080/08858190109528778	Hill et al. (2) [26], Mustermann et al. (9) [27]
Shabaik S et al. (2010) Colorectal cancer video for the deaf community: a randomized control trial. J Cancer Educ 25(4):518–523. https://doi.org/10.1007/s13187-010-0113-y	Hill et al. (2) [26], Mustermann et al. (9) [27]
Yao CS et al. (2012) Cervical cancer control: deaf and hearing women’s response to an educational video. J Cancer Educ 27(1):62–66. https://doi.org/10.1007/s13187-011-0264-5	Hill et al. (2) [26], Mustermann et al. (9) [27]
Zazove P et al. (2012) Effectiveness of videos improving cancer prevention knowledge in people with profound hearing loss. J Cancer Educ 27(2):327–337. https://doi.org/10.1007/s13187-011-0292-1	Hill et al. (2) [26], Mustermann et al. (9) [27]
