# Supplementary material for: Interventions Addressing Health Literacy in Cancer Care: A Systematic Review of Reviews
Source: Int J Environ Res Public Health. 2025 Feb 2;22(2):212. doi: 10.3390/ijerph22020212 (PMC11855911; doi:10.3390/ijerph22020212)
Supplement: Supplementary file 1 [file ijerph-22-00212-s001.zip › ijerph-3443345-supplementary.pdf]

Supplementary Material: Keywords for Systematic Review of Reviews

Interventions addressing Health Literacy in Cancer Care: A systematic review of reviews

EMBASE

('health literacy'/exp OR 'information literacy'/exp OR 'data literacy'/exp OR 'comprehension'/exp OR 'understanding'/exp OR 'information seeking'/exp OR 'knowledge acquisition'/exp OR 'ehealth literacy'/exp OR 'online health literacy'/exp OR 'e-health literacy'/exp) AND ('health promotion'/exp OR 'patient education'/exp OR 'health education'/exp OR 'health fairs'/exp OR 'cancer education'/exp) AND ('intervention study'/exp OR 'interventional study'/exp OR 'intervention trial'/exp OR 'communication'/exp OR 'interpersonal communication'/exp OR 'teach-back communication'/exp) AND ('adult'/exp OR 'aged'/exp OR 'elderly'/exp OR 'senior citizen'/exp OR 'healthcare worker'/exp OR 'nurse'/exp) AND ('oncology'/exp OR 'cancer'/exp OR 'neoplasm'/exp OR 'malignant tumor'/exp) AND ('systematic review'/exp OR 'meta-analysis'/exp)

SCIENCEDIRECT

TITLE-ABS-KEY(("health literacy" OR "health literacy" OR "information literacy" OR "data literacy" OR "information literacy" OR "comprehension" OR "comprehension" OR "passive comprehension" OR "understanding" OR "information seeking" OR "information seeking" OR "information seeking behavior" OR "information seeking behaviour" OR "learning"/de OR "knowledge acquisition" OR "ehealth literacy" OR "online health literacy" OR "e-health literacy" OR "ehealth literacy")) AND TITLE-ABS-KEY(("health promotion" OR "health promotion" OR "healthy people 2010" OR "healthy people programs" OR "promotion, health" OR "patient education" OR "education, patient" OR "patient education" OR "patient education as topic" OR "patient medication knowledge" OR "psychoeducation" OR "psychoeducation" OR "workshop" OR "shop, work" OR "workshop" OR "health education" OR "education, health" OR "health education" OR "health fairs" OR "health science education" OR "health sciences education" OR "cancer education")) AND TITLE-ABS-KEY(("intervention study" OR "intervention studies" OR "intervention study" OR "intervention trial" OR "interventional studies" OR "interventional study" OR "interventional trial" OR "intervention" OR "interpersonal communication" OR "communication" OR "communication (interpersonal)" OR "disclosure" OR "interpersonal communication" OR "teach-back communication" OR "truth disclosure" OR "doctor patient relationship" OR "bedside psychology" OR "doctor patient contact" OR "doctor patient relation" OR "professional patient relationship" OR "doctor patient relationship" OR "hospital patient relationship" OR "hospital-patient relations" OR "patient doctor relation" OR "patient doctor relationship" OR "patient physician relation" OR "patient physician relationship" OR "patient staff relation" OR "patient therapist relation" OR "patient therapist relationship" OR "physician patient relation" OR "physician patient relationship" OR "physician-patient relations" OR "psychology, bedside" OR "relation, doctor patient" OR "therapeutic relation" OR "therapist patient relation" OR "therapist patient relationship" OR "doctor patient communication" OR "cancer education" OR intervention OR "doctor patient communication")) AND TITLE-ABS-KEY(("adult" OR "adult" OR "adults" OR "grown-ups" OR "grownup" OR "grownups" OR "aged" OR "aged" OR "aged patient" OR "aged people" OR "aged person" OR "aged subject" OR "elderly" OR "elderly patient" OR "elderly people" OR "elderly person" OR "elderly subject" OR "senior citizen" OR "senium"

OR "health care personnel" OR "health care personnel" OR "health care practitioner" OR "health care professional" OR "health care provider" OR "health care worker" OR "health personnel" OR "health profession personnel" OR "health worker" OR "healthcare personnel" OR "healthcare practitioner" OR "healthcare professional" OR "healthcare provider" OR "healthcare worker" OR "public health officer" OR "hospital personnel" OR "hospital employee" OR "hospital personnel" OR "hospital staff" OR "hospital staffing" OR "hospital worker" OR "personnel, hospital" OR "health auxiliary" OR "auxiliary health worker" OR "community health aide/practitioner" OR "community health practitioner" OR "health auxiliary" OR "medical auxiliary" OR "clinician" OR "clinician" OR "clinicians" OR "medical personnel" OR "district medical officer" OR "medical officer" OR "medical personnel" OR "medical worker" OR "nurse" OR "nurse" OR "nurses" OR "nursing assistance" OR "oncology nursing"/de OR "cancer nursing" OR "oncologic nursing" OR "oncology nursing" OR "palliative nursing" OR "hospice and palliative care nursing" OR "palliative care nursing" OR "palliative nursing")) AND TITLE-ABS-KEY(("oncology" OR "cancerology" OR "clinical oncology" OR "medical oncology" OR "oncology" OR "neoplasm" OR "acral tumor" OR "acral tumour" OR "embryonal and mixed neoplasms" OR "germ cell and embryonal neoplasms" OR "glandular and epithelial neoplasms" OR "hormone-dependent neoplasms" OR "neoplasia" OR "neoplasm" OR "neoplasms" OR "neoplastic disease" OR "neoplastic entity" OR "neoplastic mass" OR "post-traumatic neoplasms" OR "tumor" OR "tumoral entity" OR "tumoral mass" OR "tumorous entity" OR "tumorous mass" OR "tumour" OR "tumoural entity" OR "tumoural mass" OR "tumourous entity" OR "tumourous mass" OR "carcinoma" OR "carcinoma" OR "carcinoma 63" OR "carcinoma, krebs 2" OR "carcinoma, scirrhous" OR "epithelial carcinoma" OR "epithelial malignant tumor" OR "epithelial malignant tumour" OR "internal carcinoma" OR "malignant epithelial tumor" OR "malignant epithelial tumour" OR "microcarcinoma" OR "neoplasm, malignant epithelial" OR "primary carcinoma" OR "hematology" OR "haematologic problem" OR "haematology" OR "hematologic problem" OR "hematology" OR "hemography" OR "malignant neoplasm" OR "cancer" OR "cancers" OR "malignant neoplasia" OR "malignant neoplasm" OR "malignant neoplastic disease" OR "malignant tumor" OR "malignant tumour" OR "neoplasia, malignant" OR "tumor, malignant" OR "tumour, malignant" OR "cancer patient" OR "cancer patient" OR "cancer sufferer" OR "cancer sufferers")) AND TITLE-ABS-KEY(("systematic review" OR "systematic"))

PUBMED

("health literacy"[MeSH Terms] OR "information literacy"[MeSH Terms] OR "comprehension"[MeSH Terms] OR "information seeking behavior"[MeSH Terms] OR "learning"[MeSH Terms] OR "health literacy"[Title/Abstract] OR "information literacy"[Title/Abstract] OR "data literacy"[Title/Abstract] OR "access to information"[Title/Abstract] OR "comprehension"[Title/Abstract] OR "passive comprehension"[Title/Abstract] OR "understanding"[Title/Abstract] OR "information seeking"[Title/Abstract] OR "information seeking behavior"[Title/Abstract] OR "information seeking behaviour"[Title/Abstract] OR "learning"[Title/Abstract] OR "knowledge acquisition"[Title/Abstract] OR "online health literacy"[Title/Abstract] OR "e-health literacy"[Title/Abstract] OR "ehealth literacy"[Title/Abstract])
AND ("health promotion"[MeSH Terms] OR "patient education as topic"[MeSH Terms] OR "health education"[MeSH Terms] OR "health promotion"[Title/Abstract] OR "healthy people 2010"[Title/Abstract] OR "healthy people programs"[Title/Abstract] OR "healthy people programs"[MeSH Terms] OR "promotion, health"[Title/Abstract] OR "patient education"[Title/Abstract] OR "patient education as topic"[Title/Abstract] OR "patient medication knowledge"[Title/Abstract] OR "psychoeducation"[Title/Abstract] OR "health

education"[Title/Abstract] OR "workshop"[Title/Abstract] OR "health fairs"[Title/Abstract] OR "health science education"[Title/Abstract] OR "health sciences education"[Title/Abstract] OR "cancer education"[Title/Abstract])
AND ("communication"[MeSH Terms] OR "health communication"[MeSH Terms] OR "professional patient relations"[MeSH Terms] OR "intervention studies"[Title/Abstract] OR "intervention study"[Title/Abstract] OR "intervention trial"[Title/Abstract] OR "interventional studies"[Title/Abstract] OR "interventional study"[Title/Abstract] OR "interventional trial"[Title/Abstract] OR "intervention"[Title/Abstract] OR "interpersonal communication"[Title/Abstract] OR "communication"[Title/Abstract] OR "communication (interpersonal)"[Title/Abstract] OR "disclosure"[Title/Abstract] OR "interpersonal communication"[Title/Abstract] OR "teach-back communication"[Title/Abstract] OR "truth disclosure"[Title/Abstract] OR "doctor patient relationship"[Title/Abstract] OR "bedside psychology"[Title/Abstract] OR "doctor patient contact"[Title/Abstract] OR "doctor patient relation"[Title/Abstract] OR "doctor patient relationship"[Title/Abstract] OR "hospital patient relationship"[Title/Abstract] OR "hospital-patient relations"[Title/Abstract] OR "patient doctor relation"[Title/Abstract] OR "patient doctor relationship"[Title/Abstract] OR "professional patient relation*"[Title/Abstract] OR "patient physician relation"[Title/Abstract] OR "patient physician relationship"[Title/Abstract] OR "patient staff relation"[Title/Abstract] OR "patient therapist relation"[Title/Abstract] OR "patient therapist relationship"[Title/Abstract] OR "physician patient relation"[Title/Abstract] OR "physician patient relationship"[Title/Abstract] OR "physician-patient relations"[Title/Abstract] OR "psychology, bedside"[Title/Abstract] OR "relation, doctor patient"[Title/Abstract] OR "therapeutic relation"[Title/Abstract] OR "therapist patient relation"[Title/Abstract] OR "therapist patient relationship"[Title/Abstract] OR "doctor patient communication"[Title/Abstract] OR "cancer education"[Title/Abstract] OR intervention [Title/Abstract] OR "doctor patient communication"[Title/Abstract])
AND ("adult"[MeSH Terms] OR "young adult"[MeSH Terms] OR "middle aged"[MeSH Terms] OR "aged"[MeSH Terms] OR "health personnel"[MeSH Terms] OR "adult"[Title/Abstract] OR "adults"[Title/Abstract] OR "grown-ups"[Title/Abstract] OR "grownup"[Title/Abstract] OR "grownups"[Title/Abstract] OR "aged"[Title/Abstract] OR "aged patient"[Title/Abstract] OR "aged people"[Title/Abstract] OR "aged person"[Title/Abstract] OR "aged subject"[Title/Abstract] OR "elderly"[Title/Abstract] OR "elderly patient"[Title/Abstract] OR "elderly people"[Title/Abstract] OR "elderly person"[Title/Abstract] OR "elderly subject"[Title/Abstract] OR "senior citizen"[Title/Abstract] OR "senium"[Title/Abstract] OR "health care personnel"[Title/Abstract] OR "health care practitioner"[Title/Abstract] OR "health care professional"[Title/Abstract] OR "health care provider"[Title/Abstract] OR "health care worker"[Title/Abstract] OR "health personnel"[Title/Abstract] OR "health profession personnel"[Title/Abstract] OR "health worker"[Title/Abstract] OR "healthcare personnel"[Title/Abstract] OR "healthcare practitioner"[Title/Abstract] OR "healthcare professional"[Title/Abstract] OR "healthcare provider"[Title/Abstract] OR "healthcare worker"[Title/Abstract] OR "public health officer"[Title/Abstract] OR "hospital personnel"[Title/Abstract] OR "hospital employee"[Title/Abstract] OR "hospital personnel"[Title/Abstract] OR "hospital staff"[Title/Abstract] OR "hospital staffing"[Title/Abstract] OR "hospital worker"[Title/Abstract] OR "personnel, hospital"[Title/Abstract] OR "health auxiliary"[Title/Abstract] OR "auxiliary health worker"[Title/Abstract] OR "community health aide/practitioner"[Title/Abstract] OR "community health practitioner"[Title/Abstract] OR "health auxiliary"[Title/Abstract] OR "medical auxiliary"[Title/Abstract] OR "clinician"[Title/Abstract] OR "clinicians"[Title/Abstract] OR "medical personnel"[Title/Abstract] OR "district medical

officer"[Title/Abstract] OR "medical officer"[Title/Abstract] OR "medical personnel"[Title/Abstract] OR "medical worker"[Title/Abstract] OR "nurse"[Title/Abstract] OR "nurses"[Title/Abstract] OR "nursing assistance"[Title/Abstract] OR "oncology nursing"[Title/Abstract] OR "cancer nursing"[Title/Abstract] OR "oncologic nursing"[Title/Abstract] OR "palliative nursing"[Title/Abstract] OR "hospice and palliative care nursing"[Title/Abstract] OR "palliative care nursing"[Title/Abstract])
AND ("neoplasms"[MeSH Terms] OR "hematology"[MeSH Terms] OR "oncology"[Title/Abstract] OR "cancerology"[Title/Abstract] OR "clinical oncology"[Title/Abstract] OR "medical oncology"[Title/Abstract] OR "oncology"[Title/Abstract] OR "neoplasm"[Title/Abstract] OR "acral tumor"[Title/Abstract] OR "acral tumour"[Title/Abstract] OR "embryonal and mixed neoplasms"[Title/Abstract] OR "germ cell and embryonal neoplasms"[Title/Abstract] OR "glandular and epithelial neoplasms"[Title/Abstract] OR "hormone-dependent neoplasms"[Title/Abstract] OR "neoplasia"[Title/Abstract] OR "neoplasm"[Title/Abstract] OR "neoplasms"[Title/Abstract] OR "neoplastic disease"[Title/Abstract] OR "neoplastic entity"[Title/Abstract] OR "neoplastic mass"[Title/Abstract] OR "post-traumatic neoplasms"[Title/Abstract] OR "tumor"[Title/Abstract] OR "tumoral entity"[Title/Abstract] OR "tumoral mass"[Title/Abstract] OR "tumorous entity"[Title/Abstract] OR "tumorous mass"[Title/Abstract] OR "tumour"[Title/Abstract] OR "tumoural entity"[Title/Abstract] OR "tumoural mass"[Title/Abstract] OR "tumourous entity"[Title/Abstract] OR "tumourous mass"[Title/Abstract] OR "carcinoma"[Title/Abstract] OR "carcinoma*"[Title/Abstract] OR "epithelial malignant tumor"[Title/Abstract] OR "epithelial malignant tumour"[Title/Abstract] OR "internal carcinoma"[Title/Abstract] OR "malignant epithelial tumor"[Title/Abstract] OR "malignant epithelial tumour"[Title/Abstract] OR "microcarcinoma"[Title/Abstract] OR "neoplasm, malignant epithelial"[Title/Abstract] OR "primary carcinoma"[Title/Abstract] OR "hematology"[Title/Abstract] OR "haematologic problem"[Title/Abstract] OR "haematology"[Title/Abstract] OR "hematologic problem"[Title/Abstract] OR "hematology"[Title/Abstract] OR "hemography"[Title/Abstract] OR "malignant neoplasm"[Title/Abstract] OR "cancer"[Title/Abstract] OR "cancers"[Title/Abstract] OR "malignant neoplasia"[Title/Abstract] OR "malignant neoplasm"[Title/Abstract] OR "malignant neoplastic disease"[Title/Abstract] OR "malignant tumor"[Title/Abstract] OR "malignant tumour"[Title/Abstract] OR "neoplasia, malignant"[Title/Abstract] OR "tumor, malignant"[Title/Abstract] OR "tumour, malignant"[Title/Abstract] OR "cancer patient"[Title/Abstract] OR "cancer patient"[Title/Abstract] OR "cancer sufferer"[Title/Abstract] OR "cancer sufferers"[Title/Abstract]) AND ("Systematic Review"[Publication Type] OR systematic[Title])

PSYCINFO

(MAINSUBJECT.EXACT("Health Literacy") OR MAINSUBJECT.EXACT("Active Living") OR MAINSUBJECT.EXACT("Health Maintenance Organizations") OR MAINSUBJECT.EXACT("Health Screening") OR MAINSUBJECT.EXACT("Health Behavior") OR MAINSUBJECT.EXACT("Health Attitudes") OR MAINSUBJECT.EXACT("Health Knowledge") OR MAINSUBJECT.EXACT("Health Behavior Measures") OR MAINSUBJECT.EXACT("Cancer Screening") OR MAINSUBJECT.EXACT("Population Health") OR MAINSUBJECT.EXACT("Health Awareness") OR MAINSUBJECT.EXACT("Information Literacy") OR MAINSUBJECT.EXACT("Information Seeking") OR MAINSUBJECT.EXACT("Exploratory Behavior") OR

MAINSUBJECT.EXACT("Information Literacy") OR MAINSUBJECT.EXACT("Information Seeking") OR MAINSUBJECT.EXACT("Health Information") OR MAINSUBJECT.EXACT("Adaptive Learning") OR MAINSUBJECT.EXACT("Learning") OR TI("health literacy" OR "information literacy" OR "data literacy" OR "access to information" OR "comprehension" OR "passive comprehension" OR "understanding" OR "information seeking" OR "information seeking behavior" OR "information seeking behaviour" OR "learning" OR "knowledge acquisition" OR "online health literacy" OR "e-health literacy" OR "ehealth literacy") OR AB("health literacy" OR "information literacy" OR "data literacy" OR "access to information" OR "comprehension" OR "passive comprehension" OR "understanding" OR "information seeking" OR "information seeking behavior" OR "information seeking behaviour" OR "learning" OR "knowledge acquisition" OR "online health literacy" OR "e-health literacy" OR "ehealth literacy")) AND (MAINSUBJECT.EXACT("Health Promotion") OR MAINSUBJECT.EXACT("Client Education") OR MAINSUBJECT.EXACT("Health Education") OR TI("health promotion" OR "healthy people 2010" OR "healthy people program*" OR "promotion, health" OR "patient education" OR "patient education as topic" OR "patient medication knowledge" OR "psychoeducation" OR "health education" OR "workshop" OR "health fairs" OR "health science education" OR "health sciences education" OR "cancer education") OR AB("health promotion" OR "healthy people 2010" OR "healthy people program*" OR "promotion, health" OR "patient education" OR "patient education as topic" OR "patient medication knowledge" OR "psychoeducation" OR "health education" OR "workshop" OR "health fairs" OR "health science education" OR "health sciences education" OR "cancer education")) AND (MAINSUBJECT.EXACT("Communication Skills Training") OR MAINSUBJECT.EXACT("Information") OR MAINSUBJECT.EXACT("Communication") OR MAINSUBJECT.EXACT("Knowledge Transfer") OR MAINSUBJECT.EXACT("Communication Skills") OR MAINSUBJECT.EXACT("Health Information") OR TI("communication" OR "health communication" OR "professional patient relations" OR "intervention studies" OR "intervention study" OR "intervention trial" OR "interventional studies" OR "interventional study" OR "interventional trial" OR "intervention" OR "interpersonal communication" OR "communication" OR "communication (interpersonal)" OR "disclosure" OR "interpersonal communication" OR "teach-back communication" OR "truth disclosure" OR "doctor patient relationship" OR "bedside psychology" OR "doctor patient contact" OR "doctor patient relation" OR "doctor patient relationship" OR "hospital patient relationship" OR "hospital-patient relations" OR "patient doctor relation" OR "patient doctor relationship" OR "professional patient relation*" OR "patient physician relation" OR "patient physician relationship" OR "patient staff relation" OR "patient therapist relation" OR "patient therapist relationship" OR "physician patient relation" OR "physician patient relationship" OR "physician-patient relations" OR "psychology, bedside" OR "relation, doctor patient" OR "therapeutic relation" OR "therapist patient relation" OR "therapist patient relationship" OR "doctor patient communication" OR "cancer education" OR intervention OR "doctor patient communication") OR AB("communication" OR "health communication" OR "professional patient relations" OR "intervention studies" OR "intervention study" OR "intervention trial" OR "interventional studies" OR "interventional study" OR "interventional trial" OR "intervention" OR "interpersonal communication" OR "communication" OR "communication (interpersonal)" OR "disclosure" OR "interpersonal communication" OR "teach-back communication" OR "truth disclosure" OR "doctor patient relationship" OR "bedside psychology" OR "doctor patient contact" OR "doctor patient relation" OR "doctor patient relationship" OR "hospital patient relationship" OR "hospital-patient relations" OR "patient doctor relation" OR "patient doctor relationship" OR "professional patient relation*" OR "patient physician relation" OR "patient physician

relationship" OR "patient staff relation" OR "patient therapist relation" OR "patient therapist relationship" OR "physician patient relation" OR "physician patient relationship" OR "physician-patient relations" OR "psychology, bedside" OR "relation, doctor patient" OR "therapeutic relation" OR "therapist patient relation" OR "therapist patient relationship" OR "doctor patient communication" OR "cancer education" OR intervention OR "doctor patient communication")) AND (MAINSUBJECT.EXACT("Middle Adulthood") OR MAINSUBJECT.EXACT("Older Adulthood") OR MAINSUBJECT.EXACT("Emerging Adulthood") OR MAINSUBJECT.EXACT("Rescue Workers") OR MAINSUBJECT.EXACT("Teleconsultation") OR MAINSUBJECT.EXACT("Counselors") OR MAINSUBJECT.EXACT("Professional Measures") OR MAINSUBJECT.EXACT("Frontline Employees") OR MAINSUBJECT.EXACT("Therapists") OR MAINSUBJECT.EXACT("Health Personnel") OR MAINSUBJECT.EXACT("Social Workers") OR TI("adult" OR "adults" OR "grown-ups" OR "grownup" OR "grownups" OR "aged" OR "aged patient" OR "aged people" OR "aged person" OR "aged subject" OR "elderly" OR "elderly patient" OR "elderly people" OR "elderly person" OR "elderly subject" OR "senior citizen" OR "senium" OR "health care personnel" OR "health care practitioner" OR "health care professional" OR "health care provider" OR "health care worker" OR "health personnel" OR "health profession personnel" OR "health worker" OR "healthcare personnel" OR "healthcare practitioner" OR "healthcare professional" OR "healthcare provider" OR "healthcare worker" OR "public health officer" OR "hospital personnel" OR "hospital employee" OR "hospital personnel" OR "hospital staff" OR "hospital staffing" OR "hospital worker" OR "personnel, hospital" OR "health auxiliary" OR "auxiliary health worker" OR "community health aide/practitioner" OR "community health practitioner" OR "health auxiliary" OR "medical auxiliary" OR "clinician" OR "clinicians" OR "medical personnel" OR "district medical officer" OR "medical officer" OR "medical personnel" OR "medical worker" OR "nurse" OR "nurses" OR "nursing assistance" OR "oncology nursing" OR "cancer nursing" OR "oncologic nursing" OR "palliative nursing" OR "hospice and palliative care nursing" OR "palliative care nursing") OR AB("adult" OR "adults" OR "grown-ups" OR "grownup" OR "grownups" OR "aged" OR "aged patient" OR "aged people" OR "aged person" OR "aged subject" OR "elderly" OR "elderly patient" OR "elderly people" OR "elderly person" OR "elderly subject" OR "senior citizen" OR "senium" OR "health care personnel" OR "health care practitioner" OR "health care professional" OR "health care provider" OR "health care worker" OR "health personnel" OR "health profession personnel" OR "health worker" OR "healthcare personnel" OR "healthcare practitioner" OR "healthcare professional" OR "healthcare provider" OR "healthcare worker" OR "public health officer" OR "hospital personnel" OR "hospital employee" OR "hospital personnel" OR "hospital staff" OR "hospital staffing" OR "hospital worker" OR "personnel, hospital" OR "health auxiliary" OR "auxiliary health worker" OR "community health aide/practitioner" OR "community health practitioner" OR "health auxiliary" OR "medical auxiliary" OR "clinician" OR "clinicians" OR "medical personnel" OR "district medical officer" OR "medical officer" OR "medical personnel" OR "medical worker" OR "nurse" OR "nurses" OR "nursing assistance" OR "oncology nursing" OR "cancer nursing" OR "oncologic nursing" OR "palliative nursing" OR "hospice and palliative care nursing" OR "palliative care nursing")) AND (MAINSUBJECT.EXACT("Neoplasms") OR TI("oncology" OR "cancer*" OR "clinical oncology" OR "medical oncology" OR "oncology" OR "neoplasm" OR "acral tumor" OR "acral tumour" OR "embryonal and mixed neoplasms" OR "germ cell and embryonal neoplasms" OR "glandular and epithelial neoplasms" OR "hormone-dependent neoplasms" OR "neoplasia" OR "neoplasm" OR "neoplasms" OR "neoplastic disease" OR "neoplastic entity" OR "neoplastic mass" OR "post-traumatic neoplasms" OR "tumor" OR "tumoral entity" OR "tumoral mass" OR "tumorous entity" OR "tumorous mass" OR "tumour" OR

"tumoural entity" OR "tumoural mass" OR "tumourous entity" OR "tumourous mass" OR "carcinoma" OR "carcinoma*" OR "epithelial malignant tumor" OR "epithelial malignant tumour" OR "internal carcinoma" OR "malignant epithelial tumor" OR "malignant epithelial tumour" OR "microcarcinoma" OR "neoplasm, malignant epithelial" OR "primary carcinoma" OR "hematology" OR "haematologic problem" OR "haematology" OR "hematologic problem" OR "hematology" OR "hemography" OR "malignant neoplasm" OR "cancer" OR "cancers" OR "malignant neoplasia" OR "malignant neoplasm" OR "malignant neoplastic disease" OR "malignant tumor" OR "malignant tumour" OR "neoplasia, malignant" OR "tumor, malignant" OR "tumour, malignant" OR "cancer patient" OR "cancer patient" OR "cancer sufferer" OR "cancer sufferers") OR AB("oncology" OR "cancer*" OR "clinical oncology" OR "medical oncology" OR "oncology" OR "neoplasm" OR "acral tumor" OR "acral tumour" OR "embryonal and mixed neoplasms" OR "germ cell and embryonal neoplasms" OR "glandular and epithelial neoplasms" OR "hormone-dependent neoplasms" OR "neoplasia" OR "neoplasm" OR "neoplasms" OR "neoplastic disease" OR "neoplastic entity" OR "neoplastic mass" OR "post-traumatic neoplasms" OR "tumor" OR "tumoral entity" OR "tumoral mass" OR "tumorous entity" OR "tumorous mass" OR "tumour" OR "tumoural entity" OR "tumoural mass" OR "tumourous entity" OR "tumourous mass" OR "carcinoma" OR "carcinoma*" OR "epithelial malignant tumor" OR "epithelial malignant tumour" OR "internal carcinoma" OR "malignant epithelial tumor" OR "malignant epithelial tumour" OR "microcarcinoma" OR "neoplasm, malignant epithelial" OR "primary carcinoma" OR "hematology" OR "haematologic problem" OR "haematology" OR "hematologic problem" OR "hematology" OR "hemography" OR "malignant neoplasm" OR "cancer" OR "cancers" OR "malignant neoplasia" OR "malignant neoplasm" OR "malignant neoplastic disease" OR "malignant tumor" OR "malignant tumour" OR "neoplasia, malignant" OR "tumor, malignant" OR "tumour, malignant" OR "cancer patient" OR "cancer patient" OR "cancer sufferer" OR "cancer sufferers") AND (PT.EXACT("Systematic Review") OR PT.EXACT("Meta-Analysis"))
